# Patient acceptability of larval therapy for leg ulcer treatment: a randomised survey to inform the sample size calculation of a randomised trial

**DOI:** 10.1186/1471-2288-6-43

**Published:** 2006-09-01

**Authors:** ES Petherick, S O'Meara, K Spilsbury, CP Iglesias, EA Nelson, DJ Torgerson

**Affiliations:** 1 Centre for Evidence Based Nursing, Department of Health Sciences, University of York, Heslington, YO10 5DD, UK; 2 Department of Health Sciences, University of York, Heslington, YO10 5DD, UK; 3 York Trials Unit, Department of Health Sciences, University of York, Heslington, YO10 5DD, UK; 4 School of Healthcare, University of Leeds, Leeds, LS2 9UT, UK

## Abstract

**Background:**

A trial was commissioned to evaluate the effectiveness of larval therapy to debride and heal sloughy and necrotic venous leg ulcers. Larval therapy in the trial was to be delivered in either loose or bagged form. Researchers were concerned that resistance to larval therapy may threaten the feasibility of the trial. Additionally there was concern that the use of larval therapy may require a larger effect size in time to healing than originally proposed by the investigators.

**Methods:**

To formally evaluate patient preferences a survey using two randomly allocated, nurse administered questionnaires was undertaken. Patients were randomised to receive one of the two following questionnaires (i) preferences between loose larvae and standard treatment (hydrogel) or (ii) patient preferences between bagged larvae and standard therapy (hydrogel). The study was undertaken in a Vascular Clinic, in an Outpatients Department of a large teaching hospital in the North of England. The sample consisted of 35 people aged 18 years and above with at least one leg ulcer of venous or mixed (venous and arterial) aetiology.

**Results:**

Approximately 25% of participants would not consider the use of larval therapy as an acceptable treatment option for leg ulcers, regardless of the method of containment.

For the patients that would consider the use of larval therapy, different preferences in healing times required to use the therapy were observed depending upon the method of containment. The median response of those participants questioned about bagged larvae found that they would be willing to use this therapy even if they were equally able to achieve healing with the use of hydrogel by 20 weeks. For those participants questioned about the use of loose larvae complete healing would have to have taken place over 17 weeks for them to choose larvae as their preferred option rather than hydrogel. This difference was not significant (p = 0.075).

**Conclusion:**

We found no evidence of widespread resistance to the utilisation of larval therapy from patients regardless of the method of larval therapy containment. These methods have the potential to inform sample size calculations where there are concerns of patient acceptability.

## Background

Whilst there is some evidence that larval treatment effectively debrides wounds (i.e. removes necrotic tissue) [1], there is no evidence from randomised trials that larval therapy accelerates the healing of chronic wounds. Consequently in 2004, the NHS Health Technology Assessment programme commissioned a pragmatic RCT to evaluate whether larval therapy will accelerate the healing of chronic necrotic venous leg ulcers [2]. Larval therapy can be delivered in two forms: loose larvae that are placed directly onto the wound or bagged larvae that are contained in a small gauze bag on the wound. It might be presumed that the latter is more acceptable to participants since there is less chance of the larvae 'escaping' from the wound area and the larvae are mainly hidden from view. However, bagged and loose larvae may not have equivalent effects on healing. Therefore it is important to test both the acceptability and the effectiveness of the two methods of delivery.

Researchers involved in the RCT [2] were concerned that participants may have an aversion to treatment by larval therapy and if this was the case, it may threaten the feasibility of research using larval therapy. In this paper we describe a survey undertaken to determine the patient acceptability of larval therapy and to elicit the effect size in time to healing required by participants to accept larval therapy in one of two delivery mechanisms to a standard therapy (a hydrogel).

A key design issue for the trialists was defining the clinically important difference in healing time between (1) larval therapy and standard treatment (hydrogel), and (2) between the two different methods of delivering larvae (bagged or loose) to be used in the sample size calculation. In the original trial application, we sought a median difference in time to healing of 6 weeks between standard treatment and larval therapy. This was based partly on clinical experience [3] and partly on the costs of larval therapy. Participants' views on the desired treatment effect were not taken into account at this point. Although there is a body of literature on eliciting patient preferences in health care [4,5] we have not identified an evaluation designed to inform the sample size of another study.

## Methods

We undertook a survey of patient preferences using randomised allocation of two questionnaires. Both questionnaires asked about preferences between larval therapy and standard treatment (hydrogel) if hydrogel dressings were to heal their ulcer in 20 weeks and the larvae were to heal their ulcer over a range of times (between 6 and 20 weeks). One questionnaire asked about preferences for loose larvae or hydrogel at a range of healing times for loose larvae, the other questionnaire asked about preferences between bagged larvae and hydrogel for a range of healing times for bagged larvae. In both cases, the healing time for hydrogel was set at 20 weeks.

Research governance and local ethics committee approval was granted for the research. All participants were asked to provide informed consent and were reassured of the confidentially and anonymity of their responses.

We randomised participants to receive the 'bagged' or 'loose' questionnaire using sealed opaque envelopes which were sequentially numbered. Envelopes were left with the outpatient clinic's receptionist. Participants were taken to a private room to discuss participation and following consent the envelope (next in sequence) was opened in the presence of the patient. In addition, the number sequence was held by a remote researcher who could check patient number with allocation. Both questionnaires were administered by a nurse researcher (KS).

Initially participants were asked if they would consider the use of larval therapy as a therapy if their nurse or doctor recommended use of them. Patient characteristics were recorded for *all *participants. If participants were unwilling to consider the use of larval therapy no further questions about the therapy were asked. For those participants who responded that they would consider the use of larval therapy further questions were asked to determine what difference in healing time relative to hydrogel they would require to utilise larval therapy or not mind which therapy they received.

Firstly participants were asked to choose which treatment that they would prefer to receive if both hydrogel and larval therapy achieved healing at 20 weeks: hydrogel, larval therapy or no preference. If the patient responded that they would prefer larval therapy or that they had no preference at this point no further questions were asked. Then in order to minimise the number of questions presented from a maximum of 15 to a maximum of 8, the second scenario presented was one in which larval therapy would heal the ulcer at 12 weeks (i.e. an 8 week difference). If on asking this question larval therapy was preferred or there was no preference, the next set of questions would ask about the preference between therapies if healing occurred at decreasing times with the use of larval therapy i.e. 19, 18, 17, 16, 15, 14 and 13 weeks. If, on the other hand, the patient did not prefer larval therapy over hydrogel with an 8 week difference in time to healing, then decreasing times to healing with the use of larval therapy were presented, i.e. healing at 11, 10, 9, 8, 7 and 6 weeks (increasing effect sizes from 9 to 14 weeks). The first time point at which the patient had no preference or chose larval therapy was then recorded and used in the analysis.

### Sample size

A total of 35 participants were included in the study allowing us to detect, with 80% power (2 sided p = 0.05), one standard deviation difference in healing times between the two modes of larval therapy.

As the data were skewed we used a Mann-Whitney U test to determine if the differences between the median times to healing derived for loose larvae versus hydrogel and bagged larvae versus hydrogel were statistically significant.

## Results

### Participants

Forty-one participants with leg ulcers, attending a Vascular Clinic, in an Outpatients Department of a large teaching hospital in the North of England, were approached to take part in the study and thirty five participants aged 18 years and above, with at least one leg ulcer of venous or mixed (venous and arterial) aetiology, agreed to take part. Eighteen participants were allocated to the bagged questionnaire and 17 to the loose larval therapy questionnaire. Table 1 shows the characteristics of the participants randomised to the different surveys.

**Table 1:** Characteristics of patients surveyed

	**Loose larvae questionnaire**	**Bagged larvae questionnaire**
Gender		
Male	8	11
Female	9	7
Mean age of participants, (SD)	73.29 years (10.09)	68.56 years (12.05)
Range	57–93 years	46–92 years
Mean duration of current ulcer (SD)	3.18 years (3.95)	3.04 years (3.1)
Range	2 months–14 years	3 months–12 years

The majority of leg ulcer participants interviewed (27/35, 77%) stated that they would consider the use of larval therapy, irrespective of the method of containment. However, almost a quarter of participants (8/35) stated that they would never consider the use of larval therapy, four people in each group. These participants were not asked any further questions about the use of larval therapy.

The difference between hydrogel and larvae healing times required for patients to choose bagged larvae over hydrogel ranged from 0 to 8 weeks, with a median value of 0, indicating that no reduction in healing time would be required for participants to choose bagged larval therapy over hydrogel. However, the difference in hydrogel and larvae healing times required for patients to chose loose larvae over hydrogel ranged from 0 to 12 weeks, with a median difference in healing times of 3 weeks, indicating that complete healing would have to be achieved in 17 weeks (median) for loose larvae, in order for this to be preferred over hydrogel.

The 3 week difference in effect sizes required to choose larvae in preference to hydrogel between the loose and the bagged larval therapy groups was not statistically significant (p = 0.075) Table 2 shows the differences between the groups of those participants who would consider larval therapy.

**Table 2:** Differences in healing times required to prefer larvae over hydrogel

	**Loose**	**Bagged**	**p-value**
Interquartile range	0–5	0–1.75	
Median	3	0	0.075
Mode	1	0	

## Discussion

We have undertaken a study of the effect sizes deemed important by participants who would consider larval therapy for leg ulcer treatment. Our study failed to find evidence of a widespread resistance to the idea of using larvae as a treatment for leg ulcers. Irrespective of the method of containment of the larvae, a similar proportion of participants (approximately three quarters) would consider the use of larval therapy. It is difficult to compare this treatment refusal rate with other studies as larvae are vastly different to other existing therapies for the treatment of leg ulcers.

No significant difference was observed in the time to healing required by patients to choose larvae, either loose or bagged, in preference to hydrogel. A possible reason for this non-significant result may be the study power, as our initial sample size calculation did not allow for 25% of participants to refuse consideration of larval therapy (clinicians estimated 10% refusal rate).

## Conclusion

Our study found that patients did not have a widespread resistance to the idea of using larval therapy for the treatment of their leg ulcers, regardless of the method of containment of the larvae. Furthermore, we found that participants were willing to have a much smaller median difference in time to healing than the six weeks proposed by clinicians, and used in the original sample size for the study. As such, researchers were able to feel confident in the patient acceptability of the intervention as the original sample size proposed greater differences in time to healing than that proposed by participants.

We found that using a relatively simple, interviewer administered questionnaire we could elicit meaningful information on the effect size for an intervention from participants. This method has the potential to elicit information to be used in sample size calculations for randomised trials in cases where there is concern over the patient acceptability of a therapy. It would be useful to repeat this exercise for interventions that may have significant drawbacks to the patient as well as potential benefits.

## Competing interests

The author(s) declare that they have no competing interests.

## Authors' contributions

SOM, CI and EAN contributed to the study protocol and questionnaire, KS conducted the interviews and collected data, ESP, SOM, CI and DJT analysed the data, ESP drafted the manuscript, SOM, KS, CI, EAN and DJT provided critical feedback on the manuscript. All authors approved the final version of the manuscript.

## Pre-publication history

The pre-publication history for this paper can be accessed here:


